# Challenges to the Poultry Industry: Current Perspectives and Strategic Future After the COVID-19 Outbreak

**DOI:** 10.3389/fvets.2020.00516

**Published:** 2020-08-26

**Authors:** Hafez M. Hafez, Youssef A. Attia

**Affiliations:** ^1^Faculty of Veterinary Medicine, Institute of Poultry Diseases, Free University Berlin, Berlin, Germany; ^2^Department of Agriculture, Faculty of Environmental Sciences, King Abdulaziz University, Jeddah, Saudi Arabia; ^3^The Strategic Center to Kingdom Vision Realization, King Abdulaziz University, Jeddah, Saudi Arabia; ^4^Animal and Poultry Production Department, Faculty of Agriculture, Damanhour University, Damanhour, Egypt

**Keywords:** poultry disease, food safety, consumer protection, Disease control, biosecurity, COVID-19, SARS-CoV-2

## Abstract

Poultry immunity, health, and production are several factors that challenge the future growth of the poultry industry. Consumer confidence, product quality and safety, types of products, and the emergence and re-emergence of diseases will continue to be major challenges to the current situation and the strategic future of the industry. Foodborne and zoonotic diseases are strictly linked with poultry. Eradication, elimination, and/or control of foodborne and zoonotic pathogens present a major challenge to the poultry industry. In addition, the public health hazards from consuming foods with high antibiotic residues will remain a critical issue. The theory of poultry production described in this review will not be limited to considering disease control. Rather, it will also incorporate the interconnection of the animals' health, welfare, and immunity. It is essential to know that chickens are not susceptible to intranasal infection by the SARS-CoV-2 (COVID-19) virus. Nevertheless, the COVID-19 pandemic will affect poultry consumption, transport, and the economics of poultry farming. It will also take into consideration economic, ethical, social dimensions, and the sustenance of the accomplishment of high environmental security. Stockholders, veterinarians, farmers, and all the partners of the chain of poultry production need to be more involved in the current situation and the strategic future of the industry to fulfill human demands and ensure sustainable agriculture. Thus, the present review explores these important tasks.

## Introduction

Disease control, high production, product quality, and reasonable production costs have been the recent main goals of the poultry industry. Hence, meeting per capita consumption and welfare to humans necessitates continuous efficient and goal-oriented healthcare to control disease spread and decrease the application of antibiotics (1). These endeavors will include the launch of programs to control infectious diseases, face the constant changes in political and social conditions, address consumers' perceptions about animal welfare, and ensure the safety and security of foods and environmental defense issues. In addition, the continuous increase in the costs of feedstuffs—and thus feeds and foods—remain prominent issues (2). The occurrence of unanticipated and new diseases and new legislation in several countries will also remain essential issues.

## Changes in Political and Social Conditions and Consumer Perceptions

### Food Safety

Consumers' perspectives on the quality and safety of animal products are a continuous issue for the poultry industry and its strategic future (3–5). Many foodborne diseases can be transferred through the food chain. In the available literature, *Salmonella* serovars and *Campylobacter* spp. are the poultry bacteria more often responsible for human foodborne diseases. In addition, public health concerns on the development of resistant bacteria due to the abuse of antibiotics as growth promoters and drugs are emerging public health challenges. Controlling zoonotic diseases and foodborne pathogens involves a deep understanding of how microbial pathogens invade and colonize, as well as the circumstances that encourage or stop growth for each strain of organism (2, 5).

The Act 2160/2003/EC was passed in November 2003 by the European Council (6) European Commission (EC, 2003) on the prevention of salmonella and other specific foodborne zoonotic agents. This directive and several protocols cover the adoption of targets aimed to decrease the occurrence of specific zoonoses at the level of principal production, in broilers, layers, and turkeys. After approval of the relevant control act, food industry workers must have samples taken and analyzed for zoonotic and zoonoses agents. In addition, a competent authority should sample the flocks. There has been a strong reduction in specific *Salmonella* serovars, including *Salmonella enteritidis* and *Salmonella typhimurium*, due to these orders (7).

*Campylobacter* spp. also contributes to foodborne diseases and represents the principal origin of zoonotic enteric infections throughout the world. *Campylobacter* infections in humans are primarily transferred via the food chain. There is no proof for either horizontal or vertical transmission from one flock to another with regard to a known poultry house contamination. However, microorganisms can be detected in the gut of killed birds. Hence, the main way for *Campylobacter* transfer in chickens seems to be the environmental horizontal transmission. The external *Campylobacter* load per bird is elevated during different slaughter processes, including transport, de-feathering, and evisceration (8). However, there are reductions during other phases of processing, with an overall 4–5 log decrease of the load from production to consumption. Adequate protocols of hygienic control should be used and strictly implemented in all phases of production. Biosecurity should be enhanced throughout the chain of production. Given that *Campylobacter* spp. can be observed in hygienic barriers, an environment should be built to maintain them far away from the poultry house (9). To guard consumers, the EU approved a unified methodology for the safety of food from the farm to the fork. The approach involves both risk management after risk measurement of assessments, including all key participants, specifically EU Member countries, the European Commission, the European Parliament, the European Food Safety Authority (EFSA), the European Centre for Disease Prevention and Control (ECDC), and economic operators. The methodology is sustained by effective and timely risk communication actions. These endeavors support European decision-makers in implementing decisions and establishing policies to safeguard consumers in the European Union (EU).

The World Health Organization (WHO) and the Food and Agriculture Organization (FAO) risk assessment definitions encompass the scientific assessment of potential adverse health and known negative effects. These concepts are vital parts of threat analysis, which comprise risk management, as well as evaluating, selecting, and applying various courses of action. These procedures should be followed by risk communication, which means exchanging information among all concerned parties.

The four steps of risk assessment are: (1) documentations of a hazard; (2) assessment of exposure; (3) hazard characterization; and (4) risk characterization (10). The “General Food Law” was established on February 21, 2002 (Regulation EC/178/2002). After a transition period, the law has been enforced since January 1, 2005 (11) to provide a framework for surveilling feeds and the health, welfare, and hygiene of animals. In addition, contaminants and residues, novel food, additives, flavorings, packaging, and irradiation of foods are covered by this law. The food law objectives are to ensure a high level of protection to human life and health, considering animal health and welfare protection, plant health, and the environment. The aims of the several pieces of EU legislation toward food safety were cited (12) as follows. First, consumer health safety is ensured by decreasing the use of medicines/antibiotics, improving resistance to disease, controlling zoonotic pathogens, and ensuring the traceability of animals and their products. Second, product safety is ensured by controlling the hygiene of the processing steps and tracing products and materials anticipated to contact the food. Third, animals should be reared and kept according to existing governmental regulations. Fourth, products and contents are improved via quality and food chain control systems and poultry and poultry-product traceability. Fifth, the tasks serve to reduce environmental contamination. Finally, rural impact, economic effects, and bio-diversity are also considered.

Other important issues are consumers' failure to apply proper, hygienic, and acceptable handling and cooking of food, as well as the limited ability of the processing plants to decrease the concentration of pathogenic microorganisms in animal products. Hence, future strategic plans are required to decrease contamination of chickens before their dispatch to processing plants and to ensure there is an adequate focus on the reduction in the availability of feed ingredients for animal feeds in view of the COVID-19 pandemic's effects on the food chain and feed industry around the world.

### Antibiotic Resistance and Related Problems

Antibiotic tolerance in humans and animals (especially bacteria) is now a common topic, and it is expected to be a continuous public health hazard (12, 13). Fortification of animals' diets with antibiotics to promote growth has increased the public's concern about the safety of animal products and their adverse effects on human health and natural immunity. The impact of antibiotics on the gut flora leads to enhanced digestion and absorption, and, thus, the availability of nutrients for production due to an improved gut ecosystem that favors beneficial microorganisms. Nonetheless, antibiotics can also amplify the occurrence of drug-tolerant bacteria. Considering the precautionary principle and experiences that have been gained in some European countries, antibiotics have been banned as “growth-promoting” for food-producing animals since January 2006. Practical opinions from experience gained in Europe revealed several problems after the ban of antibiotics in poultry nutrition: Growth and feed utilization were impaired, and ammonia level and wet litter were increased with elevated, footpad dermatitis, and thus a general decrease in animal welfare. In addition, health prospects such as enteric disorders due to clostridial infections and dysbacteriosis increased (14).

Around the world, multidrug-tolerant bacteria have progressively become a serious risk to animals and, thus, to human health and successful antibacterial treatment. Furthermore, the discovery or production of novel antibiotics does not meet the occurrence of antimicrobial tolerance in bacteria (15). For example, vancomycin-resistant enterococci (VRE) are among the multi-resistant bacteria that have increased nosocomial infections in humans (16). The prevalence of VRE in flocks of turkey raised in southwestern Germany has been investigated. Isolated enterococci were examined for the incidence of the vancomycin tolerance genes—*vanA, vanB* (B1/B2/B3), and *vanC* (C1/C2/C3)—using real-time polymerase chain reaction (PCR). VRE was observed in 15 out of 20 (75%) tested turkey flocks. Enterococci bearing van genes were also identified in dust samples (17).

Maasjost et al. (18) tested the antimicrobial sensitivity of 145 *Enterococcus* strains from poultry. Eighty-nine isolates were tolerant to three or more antimicrobial types. Turkey isolates stood out with 42 (81%) multi-tolerant isolates. The most frequent tolerant patterns of *Enterococcus faecalis* were lincomycin, tetracycline, and gentamicin in all poultry production systems.

Recently, coagulase-negative staphylococci (CoNS) were isolated from healthy turkeys in Egypt by Moawad et al. (19). All were phenotypically unaffected by tetracycline, penicillin, sulfamethoxazole/trimethoprim, and ampicillin. The tolerance rats to chloramphenicol, erythromycin, daptomycin, oxacillin, and tigecycline were 94.9, 97.4, 89.7, 92.3, and 87.2%, respectively. Thirty-one (79.5%) were impervious to linezolid. The *ermC* gene was found in all isolates tolerant to erythromycin, whereas two resistant isolates possessed three resistance-conferring genes: *ermA, ermB*, and *ermC*. The *cfr* and *optrA* genes were recorded in 11 (35.5%) and 12 (38.7%) of the 31 linezolid-resistant isolates.

Besides, livestock-connected with methicillin- resistant *Staphylococcus aureus* (LA-MRSA) has been found in a number of species of animals and farmers (20–22). Extended-spectrum beta-lactamase (ESBL) bacteria have also been observed in poultry. Richter et al. (23) examined the occurrence of LA-MRSA in turkeys' farms and farmer who reared growing turkeys. Eighteen of 20 (90%) tested flocks were confirmed to have MRSA. On 12 of the farms, 22 of the 59 (37.3%) individuals' tests were shown MRSA. None of them revealed clinical signs suggestive of a MRSA infection. The poultry farmer was expected to be positive for MRSA. Most flocks were positive for MRSA that could be allocated to clonal complex (CC) 398. In five flocks, there was MRSA of spa-type t002 that is not connected to CC398.

El-Adawy et al. (24) studied 76 *Campylobacter jejuni* isolated from the meat of 67 epidemiologically unrelated turkeys from various regions in Germany; only one isolate was sensitive to all examined antibiotics. Of the isolated, 44 (57.9%) were tolerant of amoxicillin, 69 (90.8%) to streptomycin, 61 (80.2%) to erythromycin, and 58 (76.4%) to neomycin. The tolerance to metronidazole, sulfamethoxazole/trimethoprim, nalidixic acid, ciprofloxacin, and tetracycline was 58 (76.3%), 58 (76.3%), 51 (67.1%), 53 (69.7%), and 42 (55.3%), respectively. Multidrug-resistance to three or more groups of antimicrobial substances fluctuated from 3.9 to 40.8%. Another study examined isolates gathered from various flocks of free-range turkeys in Germany (25). It reported similar results with regard to *Campylobacter* isolates to a Kenyan study that examined chickens reared in backyards and on a small scale (26).

Recently, Moawad et al. (27) reported the appearance of colistin-tolerant and extended-spectrum β-lactamase-producing *Escherichia coli* from healthy broilers in Egypt. In addition, multidrug-resistant *E. coli* has been reported from cloacal swabs of broiler chickens in Bangladesh (28). There may also be an increase in the use of antibiotics in feed-producing animals during the COVID-19 pandemic to improve animal immunity and health and increase animal farming profits. However, this use may increase the threat of antibiotic-resistant bacteria and the negative impact of antibiotics on the environment, such as cross-resistance and carryover influences.

### Welfare of Poultry

Currently, there is great concern about the welfare of animals, hygiene, and disease control that may result from great genetic pressure to boost egg and meat production. Indeed, genetic pressure to improve the productive performance of animals adversely affects animals' welfare and natural immunity and thus disease tolerance. However, genetic selection occurs with improved practices of husbandry, disease control, and nutrition manipulation (29). The most achievable alterations have been a decrease in the market age of approximately 4 weeks, a better growth rate, greater breast yield, and a higher laying rate and daily egg mass. However, there is a huge unease that the serious welfare of animals and problems of the disease have already been initiated due to the above-mentioned selection pressure. Increasing selection pressures also hinder animals' freedom (30).

Previous studies have indicated that the relationship between the pressures of genetic selection and other meteorological and husbandry factors may negatively affect animals' health status, with an emphasis on the rate of growth and bone and blood supply essential for the development of supporting structures (31). Some birds may be selected that show reduced cardiopulmonary capacity compared to traditional lines and, consequently, impaired heart and lung function. This phenomenon can result in pulmonary hypertension, sudden death syndrome, deep pectoral myopathy, and aortic rupture (32). Furthermore, genetic selection has been most implicated as the major cause of skeletal diseases, which currently draw considerable concentration as a reason for disquiet from an animal welfare point of view. The most common musculoskeletal disorders (footpad dermatitis and dyschondroplasia) are related to fast growth comprises. The incidence and severity of skeletal disorders can be affected by genetic selection and feeding (33, 34). Therefore, it is vital to know the correlation between the pressures of genetic selection and other aspects that may influence the status of animal health and disease tolerance.

Based on the literature about the new Strategy for Animal Health in the European Union (2007–2013), the theory of animal health shelters includes a lack of animal diseases and the relationship among the animals' health, welfare, and hygiene. It also considers the economic, social, and ethical concerns, as well as the methods required to accomplish a high level of hygiene and safeguard the environment (31, 35). It is expected that COVID-19 lockdowns will reduce animal welfare due to a shortage of workers for daily animal care and farming, rearing restrictions under free-range and backyard husbandry, and limited access to feeds and disease treatments.

### The Movement of Poultry and Poultry Products

Strong production competition and cost differences from around the world will affect the cost and global movement of poultry and its products. This phenomenon will increase the possibility of disease transmission into places thought to be free from poultry diseases. However, SARS-CoV-2 is not linked with poultry or its products (36, 37), it will likely influence the global poultry trade due to lockdown and restrictions that is applied to control the spread of the virus.

Globally, poultry diseases will continue to be the primary issue for the poultry industry and its strategic future. The outbreak of any disease can turn into an epidemic and have an extensive adverse influence on the global trade of poultry products. Increased feeding cost and raw ingredient prices as well their availability will negatively influence the growth of the industry and consumers' purchasing power, particularly after the COVID-19 pandemic. Moreover, increases in biogas and biofuel production will decrease the land available for grain production and feed for animal productions. This phenomenon will hinder the strategic vision of some counties, such as Saudi Arabia, to achieve their future goals. Specifically, there could be a marked increase in the cost of feeding for animal production and elevated product prices. In the future, the feed industry has an obligation to ensure the quality of feedstuffs and that they are free of pathogens and ecologically friendly. Besides, limited water resources and climatic changes are also expected to adversely affect poultry production costs and strategic planning to meet per capita consumption in areas such as Saudi Arabia (5).

### Emergence and Re-Emergence of Poultry Diseases

Several factors can hasten and/or prompt the emergence of animal diseases. These factors comprise the development and structure of the poultry farming, amplify global competition and costs of production, and increase the poultry and poultry products movement worldwide. The increased movement could also raise the hazard of introducing infections to specific regions that are free from such diseases (5). Resurgent and re-emerging infections are those that have occurred in the past but are now quickly growing either in a specific geographic area or in the host range. Infectious diseases and health disorders are mostly connected to negative economic impacts. Several pathogens are implicated as potential reasons for poultry diseases, either individually (mono-causal), in synergy with different other microorganisms (multi-causal), or facilitated by non-infectious causes.

Non-infectious agents that affect poultry health include weather conditions, hygienic status, house structure and density, water and feed hygiene, and poultry farmers' knowledge and qualifications (31). These aspects influence one another and can stimulate or control the animals' health status. Poultry farmers should provide proper nutrition, a suitable environment, husbandry, and disease control programs to ensure the desired production yield (5). Husbandry must be oriented to meet the standard rearing conditions to support optimal poultry immunity, health, and performance and to avoid disease transmission. Any stress-causing agent can hinder poultry disease resistance, increase the susceptibility of chickens to infections, and decrease the effectiveness of vaccinations.

Various infectious pathogens, including bacteria, viruses, parasites, and fungi, contribute to infectious diseases in poultry and can be transmitted and subsequently spread in farms via horizontal and/or vertical transmission (5). Just after hatching, disease transmission is mainly vertical, specifically poor hatching conditions and improper sanitation in the hatchery (omphalitis/yolk sac infection). This transmission can lead to infections with mycoplasma, aspergillus, *E. coli*, salmonella, pseudomonas, streptococci, staphylococci, encephalomyelitis, and hepatitis. The transmission of different microorganisms, including the above-mentioned one, can also occur through horizontal (lateral) means through direct contact between animals (14).

Avian flu, infectious bronchitis, Newcastle disease, Gumboro, avian metapneumovirus, *Ornithobacterium rhinotracheale, E. coli*, and mycoplasma are the most common poultry diseases around the world. In Saudi Arabia, the most common poultry diseases are avian flu, Newcastle disease, Gumboro, infectious bronchitis, epidemic tremor-avian encephalomyelitis, infectious laryngotracheitis (ILT), mycoplasma, colibacteriosis, infectious Coryza, and coccidiosis. However, these diseases depend on the hygienic conditions, geographic area, season, metrological factors, and production goals (layers vs. broilers, as well as the breed). The most hazardous disease in Saudi Arabia and many other countries is avian flu, due to a lack of appropriate vaccinations. This disease usually occurs during the winter season, with the start of migrating wild birds crossing Saudi Arabia during their route (38, 39).

Enteric disorders that result from infection by rotavirus, coronavirus enteritis, and parasitic infestation problems and *E. coli* cause substantial losses to the poultry industry. The most recent vital problems of poultry have been respiratory diseases. The severity of clinical signs, duration of disease, and rate of mortality and morbidity are highly variable and affected by virulence, type, and pathogenicity of the infectious agent(s), meteorological conditions, and environmental aspects such as high stocking density, poor management, ventilation, and condition of litter, high levels of toxic gasses such as ammonia and carbon dioxide, hygiene, coexisting diseases, and secondary infections. Animal farming around the world has become one, interconnected unit. Thus, the COVID-19 pandemic has highlighted the need for acknowledging the great risk that current viruses and future microorganisms that may lead to pandemics pose to animal health. Governments should establish new regulations for animal health care, trade, and movements of domestic and wild animals and provide adequate research funds for these activities to establish a strategic plan to ensure a continuous supply of animal protein.

### The Challenges From the SARS-CoV-2 (COVID-19) Virus

SARS-CoV-2 has emerged as systemic a zoonotic disease that poses serious hazards to humans. The Betacoronavirus group includes COVID-19, SARS-CoV, and MERS-CoV. SARS-CoV-2 is an enveloped virus that is highly infectious, even though it is easily destroyed by soap and common disinfectants. Coronaviruses are divided into alpha, beta, gamma, and delta groups. A wide range of emerging and existing diseases in food-producing animals are caused by coronaviruses (37). Various poultry body functions and systems—hepatic, renal, respiratory, neurological, and enteric—are adversely affected by coronaviruses, such as infectious bronchitis.

A prevention strategy to control the spread of COVID-19 is a lockdown, blocking transmission pathways, and educating the public to increase their awareness of the disease and decrease trade activities (40). Based on the literature, COVID-19 transmission can be impacted by some metrological factors, droplets, the population density, and direct-indirect interaction. Nevertheless, further research is required.

In order to control the recent outbreak, a global strategy must be developed and applied worldwide. The lessons that have been learned from COVID-19 are myriad and cannot be easily enumerated. However, perhaps the most important lessons are the need to boost natural immunity as the first line of defense and that the health of the world must be considered as one unit that cannot easily be disentangled. It will take time to develop an effective vaccine that can be a permanent solution, and/or specific antiviral therapy for SARS-CoV-2 (40, 41). In addition, Rahman et al. (41) called for increasing diagnostic facilities, diagnostic kits, trained physicians, and a sufficient number of hospital beds. In addition, the general public must be aware of SARS-CoV-2 to control the outbreak or break the infection cycle. In 2019, the weakness of the health system was highlighted due to an insufficient number of hospital beds, lack of adequate diagnostic kits, inadequately trained physicians, and a large number of deaths, including health professionals, physicians, nurses, and health care workers. The world must put more effort into the development of medical education, health care programs, fighting poverty, and feeding the hungry. With limited knowledge about the COVID-19 pandemic and our increasingly interconnected and multifaceted world, what is ultimately and necessarily required are robustness, flexibility, and pliability to deal with unforeseen future situations and dialogues (42).

With regard to the poultry industry, great attention has been given to restrict avian infectious bronchitis virus (IBV), which is part of the genus *Gammacoronavirus* and not transmitted to humans (37, 43, 44). Given that SARS-CoV and COVID-19 are from the same genus and use the same angiotensin-converting enzyme 2 (ACE2) host cell receptor, SARS-CoV does not infect or cause disease in poultry (39). This fact suggests that poultry is unlikely to disseminate or serve a reservoir for these viruses (36, 43). IBV induces an acute, highly contagious infectious disease of chickens (44). IBV is globally distributed and responsible for huge economic losses in the poultry industry. Initially, the virus infects the respiratory system; however, some strains show a shift in tissue tropism and spread to other organs, such as the kidneys, oviduct, and proventriculus (45, 46). The infection is mostly accompanied by a reduction in the growth rate and a drop in egg quality and quantity (47–49).

As of June 23, 2020, the mortality rate for all COVID-19 cases was 5.2% (total deaths/total confirmed cases; https://www.nsbstat.com/1/covid), which is much lower than SARS (9.6%), and far from MERS (34%), and Ebola virus (65.7%). Virus transmission is influenced by many factors, including density and movement of the population, and weather conditions such as humidity and temperature.

Although the similarity in the genetic make-up of humans and chickens is about 60%, the immune system of humans and avian species is very different, and thus vaccinations protocol, types, and applications are different. The production of human vaccines is essential to save lives and ensure the wellbeing and, hence, much more important than the production of a vaccine for farm animals. Poultry production does not seem to be at risk due to the global spread of SARS-CoV (37). Nonetheless, increasing biosecurity and hygienic measurements at the farm become apparently vital and need further efforts to limit the spread of SARS-CoV to the poultry industry and its possible mutation (50, 51). However, very limited research has been performed with the COVID-19 virus in poultry. Jackwood (37) and Swayne et al. (42) have suggested that COVID-19 is not associated with poultry or poultry products. Furthermore, recent unpublished results from the Friedrich-Loeffler-Institut [(Germany; Balkema-Buschmann et al. (36)] have shown that pigs and chickens are not susceptible to intranasal infection by SARS-CoV-2. All swab samples, as well as organ samples and contact animals, remained negative for SARS-CoV-2 RNA, while fruit bats and ferrets infected by the same route simultaneously exhibited the contrary influences which stronger in ferrets than fruit bats (Personal communication) (36, 51).

### Disease Diagnosis

The diagnosis and treatment of poultry diseases are the most common tools to control and prevent disease transmission and spread (44, 45, 52). The most recent example is avian influenza, where early diagnosis of the source and route of virus spread helped to control the disease and develop an effective vaccine for this zoonotic disease (44, 53). In a future study, improvements in laboratory diagnosis will offer sensitive, fast, and precise disease diagnosis, and early mediations will be a reality (45–47). Furthermore, vaccines developed for IBD and IBV, and early for Newcastle disease virus (NDV) and coccidiosis, have helped to save billions of dollars to the industry, as well as improve industry safety and protect it from disease outbreaks (54, 55). A major lesson from the COVID-19 pandemic is that world health is one unit. Thus, developing fast, accurate, and affordable diagenetic tools/kits that can be used on farms is absolutely necessary, and research in this area should be initiated and given maximum funding and impetus.

### Treatment

For a long time, treatment of poultry diseases has been a very successful strategy for disease control, eradication, and prevention. Good examples include bacterial, fungal, and parasitic diseases such as cholera, aflatoxin, and coccidiosis (44). Treatment of poultry diseases has allowed increased investment in the poultry industry in many developing countries that suffer from low hygiene and biosecurity (54, 55). These investments have improved and will continue to improve human welfare, fight poverty, increase family income, and ensure food security at affordable prices (2). In the future, a few authorized pharmaceuticals and veterinary products will be available to treat poultry as food-producing animals (53). Prospective research on the mechanisms of pathogenic bacteria will precisely diagnose bacterial infections, and therapy will help develop new drugs/tools that can eliminate the adverse properties of pathogens on animal's health and productivity (55). Treatment protocols for zoonotic disease and their associated secondary infections, as well as alternative protocols, should be developed after the COVID-19 pandemic.

### Disease Control

The first line of infectious disease control is to prevent the introduction of disease and to prevent further spread via strict biosecurity, establishing and maintaining immunity, and vaccination. Global poultry farming has aggressively selected for traits that focus on maximum poultry production and improving feed utilization and farming profits.

Disease control is the key element for improving animal production and food safety and, thus, human well-being. In Saudi Arabia, the National Transformation Program 2020 of the Ministry of Environment, Water and Agriculture, which is a part of the Kingdom 2030 vision, includes the following initiatives to improve animal production and to control the disease:

1. Increase the self-sufficiency ratio of broiler chickens by 42–60%;
2. Monitor infectious diseases to increase the number of animal diseases under control from 2 to 21, especially the most important and widespread diseases in the Kingdom;
3. Increase animal coverage by veterinary services from 20 to 70% to improve productivity and reduce the risk of disease and animal loss due to the cost of treatment, increased mortality, and lack of productivity;
4. Develop veterinary vaccines and production centers for local pathogenic strains;
5. Establish a program to investigate and control of infectious animal diseases.

These initiatives will hopefully succeed and positively impact animal production in Saudi Arabia to ultimately improve human wellbeing, boost self-sufficiency, and create new jobs for the younger population.

The COVID-19 pandemic has adversely impacted poultry meat production: There has been a 2% decrease in production and a 1% reduction in the global chicken meat trade in 2020 (3). These decreases may be due to lockdowns and fluctuations in global poultry supplies, feeds, shipments, meat, and eggs. The COVID-19 pandemic has highlighted the importance of poultry as a strategic, affordable animal protein. More countries must be able to achieve self-sufficiency in production, an endeavor that requires increased investment after the COVID-19 pandemic ends. Chicken meat production is expected to be decreased during the pandemic as an affordable animal protein supply that is globally accepted (4). In addition, eggs are one of the most affordable protein sources and among the most nutritious. There has been an increase in egg and poultry meat consumption during lockdowns due to several factors, such as low prices, easy and fast preparation, and high nutritional value (3, 4).

Compared to the countries with the highest meat and egg production countries, Saudi Arabia imports most of the broiler meat its citizens consume, with production accounting for less than 58% of the consumed meat (4). By contrast, it produces 17% more eggs than its needs (3). Saudi Arabia is also among the top countries in poultry meat and egg consumption. Both eggs and meat are strategic goods; thus, extended the investment in the poultry industry in Saudi Arabia is a promising agricultural sector.

The poultry meat and egg production in Saudi Arabia are presented in Table 1.

**Table 1:** Saudi Arabia meat and egg production compared with China, Brazil, and global meat (ready to cock) as well as egg number (3, 4).

**Country**	**Chicken meat production, in 1,000 metric tons**	**Percent (%) of world meat production**	**Billions of eggs**	**Percent (%) of world egg production**
Saudi Arabia	700	7.20	5.2	0.377
China	13,750	14.16	458	33.24
Brazil	13,690	14.09	53	3.85
World production	97,125	100	1,378,037,375	100

### Immunity

Pollutants, environmental, physical and physiological stress, depletion of ozone, and climate change have influenced and thus altered biological, physical, and immune functions of different animal species. Thus, animals have become less resistant to microbiota and harmful organisms. This phenomenon has adversely affected the health, welfare, and performance of animals (31). Many factors, including nutritional management, have been established to stabilize the adverse impact of environmental stressors on animal immunity and, thus, health (52–54, 56).

Immunity is the key factor in the protection of animals and disease control. The health of animals is mediated by immunity and the risk of infection and invading pathogens. Animals develop their immune system during various stages of life (55). Furthermore, animals depend on different types of immunity to protect themselves, such as natural or innate humoral immunity from limiting pathogen growth and discharging them from cells. Phagocytes (natural killer cells) are responsible for innate cellular immunity by ingesting and/or destroying pathogens and extinguishing certain cancerous cells. Innate immunity does not attack specific pathogens; rather, it works against all invading pathogens (48).

Acquired immunity (adaptive immune system) supports an efficient immune reaction after the animal's first contact with the antigen. This system enhances the animal's resistance to the same antigen in the future. Both innate and adaptive immunity work synergistically to offer animals good protection to harmful microorganisms, parasites, and other invaders. However, adaptive immunity is also implicated in allergic responses and for the rejection of transplanted tissue, which may be considered a harmful foreign invader (53).

Nutritional manipulation to boost immune functions is quite costly, but adequate nutrition is a chief factor for the advance and active effort of the immune system. Amino acids, specific fatty acids, vitamins, and minerals requirements for optimal immunity are higher than those needed for optimum growth and production performance and benefits (56). An excess of certain nutrients can harm the immunity and health of animals (57–59). The influence of dietary factors on immune function and immunity is of great interest after the COVID-19 outbreak. Currently, immunity is the first line of defense for COVID-19 until specific drugs and vaccines are available; this factor should be considered in designing strategic plans for management, feeding, and health care programs to sustain animal production.

### The Role of Nutrition

Enhancing innate immunity is the frontline of disease prevention and control. Nutrition is a key factor in immunity, disease control, and prevention. Passive and active immunity and building antibodies are affected by nutrition, quantitative and qualitative feed constituents, and hygiene. Fatty acids, protein/amino acids, minerals, and vitamins are vitally important for enhancing immunity and health (60). In general, under stress conditions, low hygiene, disease outbreaks, and the absence of effective treatment and vaccines such as the COVID-19 pandemic, enhancing protection, prevention, and control programs it is essential to improve health to ensure sustained animal performance and economic success (58, 59). Plant-based bioactive substances, antioxidants, and micronutrients are essential under the circumstances, i.e., stress and COVID-19, and have been recommended to enhance the health of the public and animals for decades in folk medicine (15, 54, 57–60). The deficiency of nutrients may increase the threat of emerging diseases such as COVID-19 and thus assuring adequate supplementation with vitamins C, E, and D is essential under such condition (61–65). Obviously, there are risk factors, such as poor nutrition and pre-existing diseases including cardiovascular diseases, obesity, diabetes mellitus, chronic lung diseases, and many others that increase patient susceptibility (62). However, nutritional intervention programs may enhance immunity and health status but have a limited impact and cannot replace advantageous clinical effects in intensive care treatment patients (66, 67).

#### Protein and Amino Acid Nutrition

The relationship between nutrition and immunity for chickens is of vital importance from a quality and quantity point of view. An important issue that might affect poultry immunity, health, and performance is dietary constituents. Crude protein is the most expensive item in poultry nutrition, at both the protein level as well as the protein source; essential amino acids are crucial (68, 69). Due to the nature of the poultry digestive tract, concentrated feeds are required, and essential amino acids rather than crude protein are vital. The use of essential amino acids may be kept at a minimum level to reduce costs and nitrogen pollution (70). Immune function is a complex system that requires higher concentrations of nutrients (amino acids, fatty acids, vitamins, and minerals) than those for productive traits. Besides, immunity is given first priority with regard to nutrient distribution among body functions. Protein and essential amino acids are vitally important for growth and antibody formation and a well-functioning immune system (71–75).

Nowadays, poultry diets are formulated based on digestible amino acids rather than crude protein. This goal is achieved by supplementation with industrial amino acids such as methionine, lysine, arginine, tryptophan, and threonine to ensure adequate intake of limiting essential amino acids (68–70). Although substantial progress has been made on ideal amino acid requirements, the optimal levels for the development of immune organs, and thus immune function, requires further investigation (73). Recent evidence has highlighted that a decrease in dietary crude protein diets could be compensated with limited amino acid supplementation at minimum crude protein level while enhancing the broilers' capacity to cope with infection (65, 70, 73). The data suggest that decreasing dietary crude protein with the same energy and essential amino acids is adequate to ensure adequate immune function in broilers (70–73). In addition, a low protein diet supplemented with essential amino acids was found to be useful tool to maintain performance and immunity of chickens when other nutrients were met (73–75).

### Fats and Fatty Acids

Essential fatty acids, principally n-3 polyunsaturated fatty acids, are essential for human and animal health and immunity. The use of n-3-fatty acids to yield functional foods impacts the nutritive value of animal products (and modulates animal and human immune function (63–65). The dietary n-6:n-3 ratio is essential for a proper immune system function. Increasing the n-6:n-3 ratio augments inflammation due to elevated pro-inflammatory mediators (cytokines), such as tumor necrosis factor α (TNF), interleukin-1 (IL-1), and IL-6. Increased cytokine levels adversely influence animal appetite (76). The effects could be attributed to T lymphocyte signaling pathways, as well as cytokine and eicosanoid synthesis (i.e., mediators of inflammatory response), and by varying the molecular configuration of lipid portfolios (64). Thus, it seems that an optimum n-6:n-3 ratio between 2–4:1 is essential to avoid an excessive inflammatory response and their resultant health hazards (63, 64). This correction can be made by using n-3-rich feeds/foods (76).

Elevating the intake of n-3 fatty acids can have harmful impacts on health, including less effective blood clotting, the production of harmful cholesterol, and altered heart rate (76–78). On the other hand, increasing n-6 fatty acid consumption increases the incidence of obesity, coronary artery diseases, and type 2 diabetes (63, 64). In humans, consumption of eicosapentaenoic acid (EPA) and docosahexaenoic acid (DHA), the fish oil n-3 fatty acids, decreases health risks from Alzheimer's disease and cardiovascular disease but impairs fetal development (76). Furthermore, DHA and EPA have a vital role in immunity modulating response (77, 78). In addition, the n-6:n-3 ratio impacts egg, meat, and milk quality and animal immunity (79). However, the nutritional requirements of essential fatty acids need further exploration. The impact of fatty acids on immunity is linked to the improved antioxidant balance that affects immune system modulation via humoral and cellular immunity (65). Lymphocyte, splenocyte, and heterophil proliferation, maturation, and cytokine production are affected by omega fatty acids. In addition, immunoglobulin G (IgG) and IgM production are increased (80–82).

Alagawany et al. (64) reviewed the most recent literature and concluded that increasing n-6 fatty acid intake concomitant with a substantial decrease in n-3 fatty acids hinders human health and immunity. They concluded it would be best to keep the n-3:n6 ratio around 1:1. Sunflower, soybean, rapeseed, and palm oils are rich sources of n-6 fatty acids, while fish and flaxseed oils and certain nuts are a good source of n-3 fatty acids—with different n-3 fatty acids such as EPA and DHA as well as alpha-linolenic acid (64). The impact of different fat sources on animal immunity and health was recently studied by Attia et al. (63), who observed that IgM and IgG were markedly elevated in animals fed a diet supplemented with canola and coconut oil compared to an unsupplemented diet. However, IgG was increased due to feeding a fish-oil-enriched diet, and fish oil and canola raised γ-globulin and α2-globulin. In addition, avian influenza and Newcastle disease antibody titers were sustainably increased due to enriching a broiler diet with 1.5% coconut fat. Fish oil supplementation increased follicle length and width of the bursa of Fabricius and depth of the thymus cortex, but the fish oil decreased the follicle length-to-width ratio. The improved immunity of broilers supplemented with canola oil concurred with an increased villus height-to-depth ratio. There was also an augmented antioxidant balance in broilers supplemented with coconut and canola oil (63).

The literature has demonstrated that the impact of oil supplementation on performance, immunity, and antioxidants depends on the criteria of response. Therefore, further research is obligatory to fully elucidate the effects of dietary oil fortification. The n-3 fatty acids addition enhances antibody production against the Newcastle disease virus (80). Fish oil supplementation has no adverse properties on the immune task of broilers (63). Additionally, fish oil markedly elevates antibody titers and the bursa of Fabricius and spleen weight and the spleen percentage (76). Birds fed a diet with a greater n-6: n-3 ratio show impaired epithelial cell growth in the intestine (79). The antioxidant balance can be corrected by the addition of natural antioxidant properties and lipid peroxidation, and immune reaction properties (80). Dietary fish oil markedly increases antibody titers and the relative spleen and bursa weights compared to the control group (81). Coconut fat ameliorates broiler digestion of lipid and raised productive performance during the course of coccidiosis infection (82).

#### Feed Additives

Nutritional immunomodulation is defined as the impact of additives on certain functions of the immune system and/or decrease hazards of infection by bacterial, viral, protozoa, and fungus. Some feed additives, such as photogenic plants, plant extract, prebiotic, probiotics, synbiotics, bee pollen and propolis, yeast, and enzymes, have reported immunomodulatory effects. Hence, there is a great interest in using them to decrease the environmental hazards and carry-over effects of antibiotics on human health (83–85). Their effects include improving metabolic status, decreasing physiological stress, inhibiting the excursion of cytokines by the macrophages, and antimicrobial activity, thus enhance immunity (86).

It is widely recognized that beneficial microbiota—probiotics, lactic acid bacteria, and *Saccharomyces cerevisiae*, with its cell wall constituents glucan and MOS—and organic acids are necessary for immunity and gut health. These additives substantially contribute to several mechanisms for disease prevention and control of pathogen growth (87). They improve antioxidants status, vitamin synthesis, and nutrients digestion (88). Beneficial microbiota can also help maintain animal health (87, 88). In this respect, the progress in the probiotic as immunomodulatory interventions shows the prospect to improve animals' tolerance to bacterial diseases such as salmonella (89), help detoxify aflatoxin (87, 88), and decrease the hazards of nitrate (90). The advantageous impact of immunobiotics on immunity and subsequent health of an animal is directly regulated by indirect a direct interrelationship (91–93). The proliferation and differentiation of cells, production of cytokines, secretion of IgA, synthesis of antimicrobial peptides, and increased intestinal cell tight junctions may be affected by the interaction activity between microorganisms and the response of both non-immune and immune cells (91–94).

Transcriptomic analyses have demonstrated that the immunomodulatory influence of immunobiotics is strain-specific (91–93). Thus, there is a need for additional studies for individual strains to explore potential immunomodulatory influence(s) (92, 93). Feed enzymes and yeast derivatives could prove to be suitable additional strategies to sustain gut function in and ensure their productive performance during coccidiosis infections (94) and under stress condition and normal condition (95, 96).

#### Minerals

The immunological effects of minerals and their essential role in immunity and health are well-known (97–99). Microelements such as calcium (Ca), phosphorus (P), and vitamin D_3_ are essential for bone health and preventing bone disease (rickets, osteomalacia, and lameness). These benefits are critical for broilers and laying hens to prevent cage layer fatigue and poor eggshell quality (100–102). Essential micro-minerals such as zinc (Zn), iron (Fe), chromium (Cr), copper (Cu), selenium (Se) and iodine (I), are important as antioxidants and immune and health enhancers, and they are required for red blood cell and thyroid hormone functions (101–104). In general, the recommended doses of trace minerals to boost immunity are ~50–100% higher than the levels needed for productive performance. This requirement depends on the type of and form of the mineral, animal age and strain, environmental stress, and hygienic conditions. However, some elements—particularly heavy metals—can have a negative effect on the environment, causing pollution due to increasing mineral excretion in animal manure (105–108).

A recent trend in mineral nutrition has focused on replacing inorganic minerals with organic sources and nanoparticles, particularly green nanoparticles obtained from plant tissues, on improving mineral utilization, animal immunity, and decrease environmental pollution (109). This method includes extracts from plants that contain proteins, sugars, terpenoids, and polyphenols, among other compounds. The reducing capacity of these phytochemicals can preserve the minerals in a reduced form during the synthetic process. Furthermore, these green nanoparticles are highly biodegradable and, therefore, do not exert negative consequences on the environment (110).

The use of nano-minerals in trace mineral nutrition increases their bioavailability, decreases antagonism between minerals at intestinal sites, and reduces excretion and, hence, trace-minerals related pollution. A recent review demonstrated that the nutritive nanoparticles enhance performance in livestock and poultry and immunity due to increased digestive effectiveness (110). Shi et al. (111) revealed that substituting FeSO_4_ with iron-glycine chelate at the identical dietary Fe concentration cannot enhance the broilers' productive criteria. However, it can effectively enhance the blood biochemical traits and the activity of the antioxidative enzyme at a 60% replacement level. Nano-forms of Cu and Zn can have a cumulative influence and may become a substitute for chelated organic and inorganic forms and can improve animal performance and immunity (110). Svetlana et al. (103) found that the degree of cutaneous hypersensitivity to phytohemagglutinin (PHA) at 21 and 35 days of age was greater in broilers given organic Fe, and Gumboro antibody titers at 35 days of age are increased in broilers offered iron chelator, but at 42 days of age, the differences are diminished among broilers.

Zn is the second most abundant microelement after iron. It has many functions, including nucleic acid synthesis and repair, metabolism, immune response, redox homeostasis, and apoptosis, and it plays a vital role in the host-pathogen relationship, and now is recommended for treatment of COVID-19 patients. Zn homeostasis is closely connected with the normal operating of both eukaryotic and prokaryotic cells, and thus many pathogens are indirectly or directly influenced by perturbations in Zn homeostasis. Zn inhibits the activity of RNA polymerase in a number of viruses, including coronavirus, hepatitis C virus, arterivirus, and rhinovirus (112–114). Immunity of poultry were improved due to Zn supplementation (112). In addition, zinc is a cofactor for the thymus hormone thymulin and modulates cytokine release and proliferation (115–117). Sajadifar et al. (98) reported that zinc has a role as a nonpharmacologic immune booster in broiler chicks. The effect of Zn on chickens' immunity is also well-documented in the literature (112). Hence, current research seeking an alternative to inorganic Zn minerals salts such as organic and nanoscale minerals. Hidayat et al. (105) reviewed the existing evidence on the effect of Zn form and concentration on immunity and found that the Zn requirement for immunity is 90.63 and 106 mg/kg diet, which is higher than that needed for growth and feed conversion ratio of broilers (93.37 and 75.72 mg/kg diet, respectively).

The trace mineral Se is essential for human and animal nutrition. It is used to sustain physiological function, immunity, health, and product quality (97–101). Se is an essential constituent of the 24 selenoproteins in the avian genome. In addition, Se is a constituent of the antioxidant enzyme glutathione peroxidase (GPX). This enzyme guards cells against oxidative stress. Se is also part of the deiodinase enzyme essential for the activation of the thyroid hormone (97, 101, 102). The main functions of selenoproteins are control of redox of biochemical function, antioxidant balance systems, thyroid hormone anabolism and catabolism (106), anticancer protection (108), and immune function booster (117, 118). Hence, Se-enhanced animal products can improve animal immunity, prevent diseases caused by Se deficiency, and enrich general health (97, 101, 102). Se fortification can enhance the antioxidant balance of animals and improve product quality and animal performance (119–121), but excess Se may hurt the environment (108, 117).

#### Vitamins

Vitamins boost animal and human immunity. Recent recommendations have been made to boost animal immunity and health under normal and heat stress conditions using vitamins: 200 mg/kg diet vitamin C, 100–200 mg/kg diet vitamin E, and 2500–4000 mg/kg diet vitamin D3, and vitamins such as vitamin C, E, and D_3_ are recommended for COVID-19 patients (121–124). Water-soluble vitamin C improves the antioxidant balance, provides antiviral function, relieves oxidative stress, enhances immunity, and spares vitamin E (125–127). α-Tocopherol, known as vitamin E, is the most common natural fat-soluble vitamin; it acts as an antioxidant that guards cell membranes against oxidative impairment due to lipid peroxidation (125–128). It also enriches the function and proliferation of lymphocytes and macrophages and increases phagocytic activity and decreases oxidative damage under normal and heat stress conditions (129, 130). Attia et al. (13, 124–126) concluded that vitamins E and C have a great effect on productive performance and immune response of animals exposed to heat stress.

Vitamin D_3_ reportedly has immunological effects that considerably influence the progress of skeletal health, muscle, and Ca and P homeostasis (131–133). Eggshell formation and bone health in laying hens is essential and involves the integration between the metabolism of Ca, P, and vitamin D_3_ (134). Vitamin D_3_, or its active form 25-(OH)D, both have strong immunomodulatory properties with the ultimate help of T cells (Th2) (131–134).

Vitamin B groups are also essential for boosting immunity due to their crucial role in the metabolism of protein, fat, and carbohydrates, and adequate vitamin B intake is essential because water-soluble vitamins are not stored in the body, and daily supply must be ensured (101, 106).

### Immune Organs

The two major avian immune system organs are the bursa of Fabricius (associated with B cells) and the thymus (associated with T cells). The bursa of Fabricius is divided into follicles; it is occupied with B lymphocytes that produce specific antibodies, which circulate in the bloodstream. B lymphocytes are initially produced in the bone marrow, embryonic liver, and yolk sac and then transferred through the blood to the bursa of Fabricius, where they mature to B lymphocytes (135). The bursa of Fabricius reaches its maximum size at 8–10 weeks of age and then undergoes regression. By 24–28 weeks of age, most of the bursa of Fabricius is heavily depleted.

The thymus comprises a paired organ located in the neck at both sides of the trachea. It includes five lobules where T cells multiply; these cells are responsible for cellular immunity (136). In addition, immune memory is recognized as a part of the lymphocytes that respond to the initial infection are preserved and may later again be activated in response to a second attack by the same microorganism. Cellular memory depends on the persistence of antigen-sensitized T cells that are able to quickly reply to secondary infection (137).

A protective immune reply to vaccination is similar to primary infection and results in the production of sensitized T lymphocytes for cellular immunity, antibodies (humoral immunity), or a combination of both processes. The mucosae comprise several defined lymphoid tissues that reply exactly to the used antigens, and this immune reaction can be either humoral or cellular (138–140).

Meteorological changes present an additional burden on immune competence and the health status of animals (135). Several investigations have shown that low ambient temperatures can reduce the feed intake and subsequently compromise immune function (141, 142).

### Vaccination

A vaccine is considered as one of the most helpful immune interventions due to its ability to encourage safeguard against infectious diseases through the targeted direction of the immune system (52). Vaccination has had and will continue to have a major influence on the development and strategic growth of the industry, allowing economic and effective control and eradication of diseases (44). However, vaccination alone without considering additional factors is not the solution for all infectious disease problems (55). In brief, the efficacy of vaccines is also influenced by many factors, including passive and active immunity, the homogeneity of the flock's immunity, immune system differences among types of birds, birds' health, metrological conditions, form, dose, and method of vaccine administration, geographic area, disease epidemiology, and cost of vaccinations. These issues will be further discussed in the next section.

Birds have several mechanisms to protect from infections and to maintain good health. Immunity is the frontline of defense and birds have several non-specific defense mechanisms such as intact skin and cilia in the respiratory system, as well as the low pH and intestinal flora in the gut system (48–50). The mucosa in the respiratory tract and digestive canals, as well as in the skin, secretes constituents such as lysozymes with vital bactericidal activity (45). During the COVID-19 pandemic, vaccine development has received great attention and has been recognized as the most effective tool for human and animal protection and disease control and eradication (48). Research to develop new vaccines and track changes in RNA viruses and disease epidemiology should continue to avoid threats from zoonotic diseases (53). In general, biosecurity, cleaning, and disinfection, as well as vaccination against some diseases, are very important for disease control and eradication; however, these programs need regular review and updating (54).

### Causes of Vaccine Breaks

In most cases, the use of vaccination creates a false sense of biosecurity and hygiene. Generally, vaccination does not prevent infection; rather, it only leads to a reduction in the number of outbreaks. Several factors can lead to the so-called vaccine break: incorrect transport and storage of the vaccines, faults or deficiencies of the administration, and high infectious pressure in an area. Additionally, incompatibilities with other control and therapy treatments such as live bacterial vaccines, using antibiotics, and/or applying live coccidiosis vaccines while simultaneously feeding animals with coccidiostat-contaminated feeds (29, 138).

Several factors can restrict the use and application of a vaccine, such as the epidemiological situation, cost-benefit analysis, availability of the vaccine, and governmental regulations. Furthermore, the following points should be considered in order to finalize the vaccination program. As a general role, adequate immunity is an obligatory need in order to manage infectious pressure on the farm, thus providing proper vaccination programs for disease control is essential to ensure the health status of poultry. Furthermore, there could be subclinical infections within the flock, and other immunosuppressive diseases or infections with field strains could occur shortly before or after the vaccination, and/or there could be infection with mutant strains. Finally, the quality of the vaccine is affected by the number of antigens, poor storage conditions, improper handling, and administration (38, 50).

The application of technologies in vaccination production, such as subunits, reverse genetics, recombinants, and nucleic acid vaccines, can considerably decrease the cost of vaccination, guarantee high efficacy, and permit rapid and easy intercession to face the fixed mutation of the microbiota. Likewise, improvement in vaccine efficiency against bacterial infections will permit a decrease in the application of antibiotics and, consequently, a reduction in antibiotic-resistant bacteria (50, 54, 55). Failure to appropriately vaccinate animals can cause a substantial reduction in poultry breeding, particularly for parental stocks; parental levels should be given considerable attention because they can directly affect maternal antibody production and transfer of passive immunity. Breeding companies should re-evaluate vaccination programs, hygiene, and biosecurity to exploit the benefits of the immune-related lesson learned from the COVID-19 pandemic in the absence of vaccines.

### Genetic Resistance

The application of selection programs to enhance health and production performance remains a long-lasting objective of the poultry industry. Shortages in the poultry industry include poor husbandry and management practices, improper nutrition and infrastructure, and above all different deadly emerging diseases that can cause enormous economic damages (55). The desire to augment breeding strategies by using molecular methods (genetic linkage maps) will lead to the characterization of genes and genome structures that are connected with production performance, disease control, and disease tolerance. This eventuality will allow the selection of lines that are genetically tolerant to some pathogens. Additionally, enhancement of poultry management, husbandry, nutrition, and rearing will help to sustain their comfort and welfare. Viral diseases such as Marek's disease, Newcastle disease, avian influenza, and bacterial diseases like salmonellosis, pasteurellosis, and colibacillosis are economic diseases that cause high mortality and morbidity (44). These diseases are candidates for the application of biotechnology tools for the selection of disease resistance (54, 55). Recent evidence has revealed that selection for disease resistance is a more successful endeavor compared with the use of vaccines or drug treatments for disease control in poultry (55). Diversity in genetic material, the immune reaction by the host animal, and the transcriptome can be employed to identify resistant genes for disease prevention. Disease resistance genes are those that encode antibodies, microRNA, and other materials that assist the host in fighting the damage caused by pathogens. Research has shown that single nucleotide polymorphisms and other candidate genes are useful tools to promote disease resistance in chickens. Additionally, studying the biological methods and genes responsible for conveying inherent resistance to different infections will lead to enhanced vaccination response to different viral and bacterial disease and/or establish disease-resistant poultry flocks (44, 55). This eventuality would decrease drug abuse and drug residues in the food chain. Thus, a good understanding of disease-resistance genes is essential. In addition, disease resistance breeding involves approaches such as genetic markers and indicator traits that can be applied for disease selection.

### Education Programs

The success of any program for disease regulation depends on hygiene, applying the proper farm sanitation, vaccination programs, and appropriate education programs for poultry farmers (2). Besides, useful education programs should be employed to markedly raise public awareness about the essential measurements required to safeguard against zoonotic diseases (54). So far, continuous education and training programs for poultry farmers have been very successful. They have enhanced and updated workers', owners', and staff members' knowledge and should continue to be employed and developed. The lessons learned from the COVID-19 pandemic indicate that public knowledge and educational levels are very important tools in public health protections and zoonosis transmission and control. These educational programs can be summarized in the following:

1. Biosecurity and hygiene in poultry farming, and immunity and disease control
2. Viral diseases and their threats and emerging diseases and their threat
3. The health of poultry farmers, Zoonotic disease control and prevention, laborers rights and welfare in poultry farms and plants
4. Basic poultry husbandry, behavior, and welfare
5. Poultry product quality and hygiene
6. A recent update on the poultry industry.

The target groups of these programs are Farm owners, workers, laborers, agriculturists, and veterinarians. In general, the expected outcomes are improve basic knowledge and scientific background related to biosecurity and hygiene of poultry framing; refresh and update relevant individuals with recent information on poultry farming, processing plants, hatcheries and feed mill; cross-react; and improve the work environment and safety. These education programs can positively impacted the industry, improved human wellbeing, and limited the loss in humans and animals (54).

## Conclusions

Global collaboration and trade will cause governments throughout the world to synchronize their current legislation linked to the market, control of disease, poultry nutrition, and the drugs and vaccines licensing for veterinary practice, particularly after the COVID-19 pandemic. The consumer desire for high-quality poultry products will robustly affect production practices. Hence, stockholders, veterinarians, farmers, and all other partners engaged in the chain of poultry industry should share more tasks and increase their cooperation. Biosecurity, hygiene, immunity are front lines of defense, perhaps the greatest lesson we learned from the COVID-19 pandemic. However, the SARS-CoV-2 (COVID-19) virus is not associated with poultry or poultry products. The poultry industry is to globally unify the health care system and biosecurity of the industry all one unit, and this lesson learned from COVID-19 as world health is one unit. Enhance protections, prevention, and control program in poultry farms. Assure product quality and imposed new programs to prevent zoonotic disease transmission. Improve the innate animal immunity as the frontline of disease prevention and control.

They were imposing strict regulations for Animal biosecurity and hygienic condition and Zoonotic diseases. Improve protections, prevention, and control disease program in poultry farms. Improve the innate animal immunity as the frontline of disease prevention and control. The emerging disease should give maximum attention. Unify and regulate global animal and poultry movement and trade of domestic and wild animals. Assure product quality and imposed new programs to prevent zoonotic disease transmission.

Considering poultry labors are frontline workers and are vitally important, not disposable, and supported them with all necessary protections such as physical and financial health to establish vital and cost-effective measures. This strategy included improving the educational background of workers through continuing education and training programs, improving biosecurity and hygienic measurements for poultrymen, slaughterhouses, and feed plants, and in farm biosecurity and hygiene.

Strategically, the COVID-19 pandemic has taught us that research must continue and be reoriented to discover new vaccines. For farms, fast and affordable diagnostic tools and supplementary methods to prevent diseases are urgently needed. The research and development in poultry disease identification and control should not be limited to currently known diseases. It should be prospective and incorporate emerging zoonotic diseases that may require new vaccines for their control. Continuous education programs should be implemented at all levels of the poultry industry and must be renewed every three years. Implementing key measures will ensure that workers' financial stability and well-being is prioritized.

## Author Contributions

All authors listed have made a substantial, direct and intellectual contribution to the work, and approved it for publication.

## Conflict of Interest

The authors declare that the research was conducted in the absence of any commercial or financial relationships that could be construed as a potential conflict of interest.
